# Adapting a Person’s Home in 3D Using a Mobile App (MapIt): Participatory Design Framework Investigating the App’s Acceptability

**DOI:** 10.2196/24669

**Published:** 2021-05-11

**Authors:** Manon Guay, Mathieu Labbé, Noémie Séguin-Tremblay, Claudine Auger, Geneviève Goyer, Emily Veloza, Natalie Chevalier, Jan Polgar, François Michaud

**Affiliations:** 1 School of Rehabilitation Faculty of Medicine and Health Sciences Sherbrooke University Sherbrooke, QC Canada; 2 Research Center on Aging Centre intégré universitaire de santé et de services sociaux de l'Estrie - Centre hospitalier universitaire de Sherbrooke Sherbrooke, QC Canada; 3 Interdisciplinary Institute for Technological Innovation Sherbrooke University Sherbrooke, QC Canada; 4 Faculty of Engineering Sherbrooke University Sherbrooke, QC Canada; 5 Center for Interdisciplinary Research in Rehabilitation of Greater Montreal Montreal, QC Canada; 6 School of Rehabilitation, Faculty of Medicine, Université de Montréal Montreal, QC Canada; 7 School of Occupational Therapy Western University London, ON Canada

**Keywords:** occupational therapy, mobile phone, aging, disability, telehealth, 3D visualization, universal design, built environment, camera, remote assessment, assistive technology

## Abstract

**Background:**

Home adaptation processes enhancing occupational engagement rely on identifying environmental barriers, generally during time-consuming home visits performed by occupational therapists (OTs). Relevance of a 3D model to the OT’s work has been attested, but a convenient and consumer-available technology to map the home environment in 3D is currently lacking. For instance, such a technology would support the exploration of home adaptations for a person with disability, with or without an OT visit.

**Objective:**

The aim of this study was to document the development and acceptability of a 3D mapping eHealth technology, optimizing its contribution to the OT’s work when conducting assessments in which home representations are essential to fit a person’s needs.

**Methods:**

A user-centered perspective, embedded in a participatory design framework where users are considered as research partners (not as just study participants), is reported. OTs, engineers, clinicians, researchers, and students, as well as the relatives of older adults contributed by providing ongoing feedback (eg, demonstrations, brainstorming, usability testing, questionnaires, prototyping). System acceptability, as per the Nielsen model, is documented by deductively integrating the data.

**Results:**

A total of 24 stakeholders contributed significantly to MapIt technology’s co-design over a span of 4 years. Fueled by the objective to enhance MapIt’s acceptability, 11 iterations lead to a mobile app to scan a room and produce its 3D model in less than 5 minutes. The app is available for smartphones and paired with computer software. Scanning, visualization, and automatic measurements are done on a smartphone equipped with a motion sensor and a camera with depth perception, and the computer software facilitates visualization, while allowing custom measurement of architectural elements directly on the 3D model. Stakeholders’ perception was favorable regarding MapIt’s acceptability, testifying to its usefulness (ie, usability and utility). Residual usability issues as well as concerns about accessibility and scan rendering still need to be addressed to foster its integration to a clinical context.

**Conclusions:**

MapIt allows to scan a room quickly and simply, providing a 3D model from images taken in real-world settings and to remotely but jointly explore home adaptations to enhance a person’s occupational engagement.

## Introduction

When the physical environment interferes with a person’s ability to do the things they want, need, or like, occupational therapists (OTs) look for potential home adaptations to enhance this person’s home and community participation. Indeed, a mismatch between a person’s capacities and the built environment might result in personal care assistance or institutionalization, increasing the financial burden for both families and health care system [1]. Carnemolla and Bridge [2] have shown an increase in health-related quality of life and well-being following a home modification process. A systematic review performed by Stark and her colleagues [3] indicates that home modifications resulted in improved function, increased ability to provide care, and decreased falls for people with a broad range of impairments.

Essential components of the home modification process for stakeholders are to jointly identify environmental barriers faced by a person to target changes and mitigate them, to add assistive technology use, and to transform occupational engagement. To do so, occupation-based intervention in the home by OTs is valued rather than relying on interviews in a remote location [4], but home visits are challenging. They are costly and time-consuming [5-7]. Moreover, OTs have reported that home visits can be stressful and anxiety provoking for some patients because it might be viewed as a “test” that they could fail [6].

Nevertheless, visual data about the architectural elements and the home design are essential for people engaged in a home adaptation process. Previous studies have investigated photography [8,9], video recording [10], and videoconference [11,12] as a substitute to a home visit but have shown mixed success. Relevance of a 3D representation for a home adaptation process has been attested by older adults [13] and OTs [14]. Some authors have reported experimenting with 3D representations for home adaptation: photogrammetry, which is a 3D construction from 2D pictures tools [15] or 3D virtual reality space design [13,14]. However, to our knowledge, available 3D drawing tools do not allow the visualization of a person’s “real” home (eg, Idapt Planning [16], OT Draw [17], Google SketchUp [18], Sweet Home 3D [19]). Some technologies create 3D scans of real-world settings but they involve either high-tech equipment [20] or remote processing of data and added sensor equipment [21].

Therefore, a convenient and consumer-available technology to map the home environment in 3D and explore adaptations with a person having disabilities without an OT visit is currently missing. This study reports on the user-centered design within an overarching participatory design process of such an eHealth technology, and on its acceptability to promote engagement of individuals facing architectural barriers in their home.

## Methods

### Framework

The choice of method to conduct MapIt’s design focuses on understanding the aspects influencing the *acceptability* of technologies [22]. It refers to the evaluation of practical and social aspects such as reliability, cost, compatibility, and usefulness (ie, usability and utility) [22,23]. Therefore, by incorporating a *user-centered perspective* [24], a *participatory design* approach was conducted, led by researchers and where users are seen as partners (not as just study participants) [25,26]. A user-centered design approach focuses on meeting the users’ needs by involving them throughout a technology’s development process [24]. It is an iterative process where the prototype is tested by users and improved according to test results, thereby fostering technology acceptance [22]. The participatory approach pushes the users’ involvement a step further by integrating some of them in the design team and having them participate in decision making during concept generation and development phases [25], to further improve the technology’s acceptability. Still, additional users, in this case clinicians, patients, and relatives, are involved during testing rounds to give a fresh look on the design. They provide the more naïve feedback valued by a user-centered design and broaden perspectives on acceptability.

### Design Process

**Figure 1:**
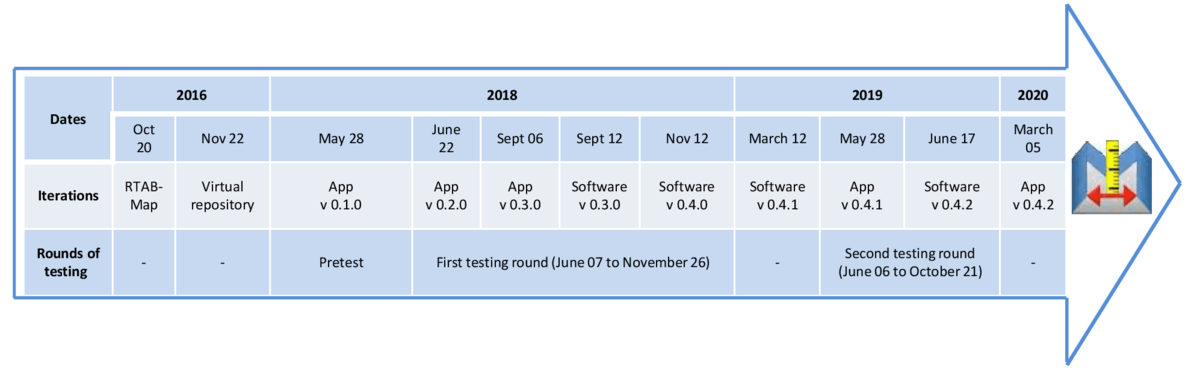
MapIt’s design process (Notes: The numbered iterations of the prototype are placed chronologically and associated to the corresponding study phase. Some version numbers are skipped to match app and software version numbers; Version 0.4.2 of the app has not yet been tested).

Figure 1 summarizes MapIt’s ongoing design process. Overall, the study was divided into 4 main phases: (1) exploration, (2) pretest, (3) first testing round, and (4) second testing round. The prototype exploration phase involved the design team envisioning the possibility to create 3D models of the home environment by using part of an existing technology. Indeed, as shown in the “Results” section, this study did not start from a blank slate. The pretest phase involved the design team creating and testing a first prototype, as well as establishing a user-centered testing protocol. Thereafter, the first and second testing rounds involved additional users to conduct multiple tests and iterations of the technology. Although the first and second testing rounds were tailored to the user-centered design approach, all 4 phases were part of a participatory design approach, as some users were part of the design team.

**Figure 2:**
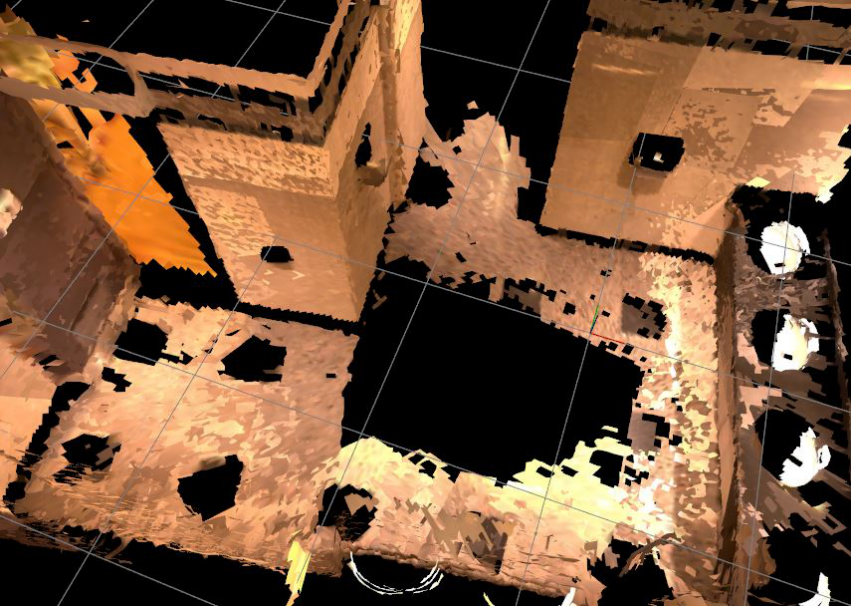
Map of a hotel bathroom to explore RTAB-Map’s possibilities.

**Figure 3:**
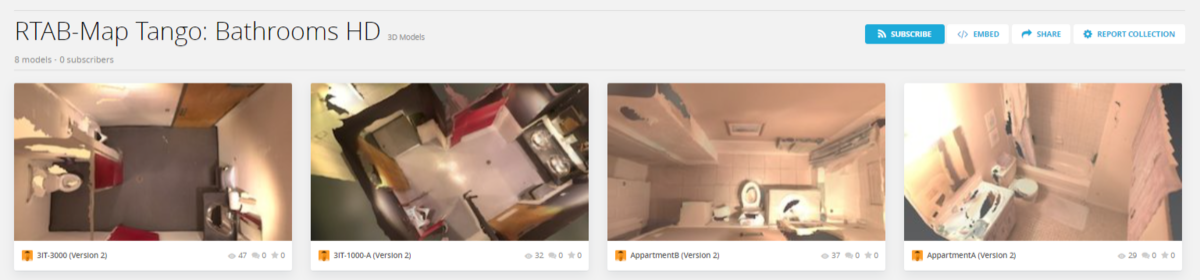
Excerpt from virtual repository on Sketchfab [28].

**Figure 4:**
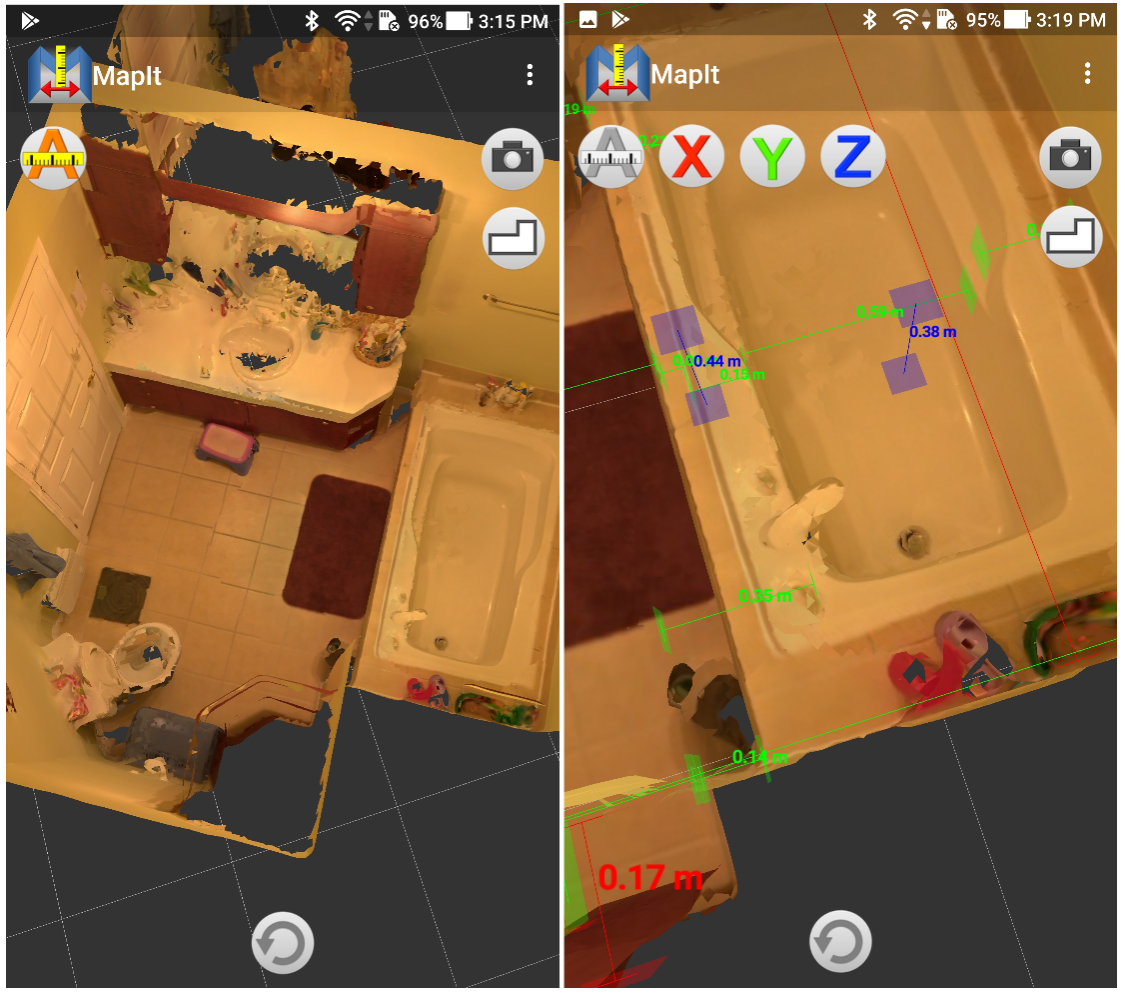
Phone app interface (V 0.4.1) when visualizing a scan. On the left, no automatic measurements were applied while on the right, automatic measurements are applied.

**Figure 5:**
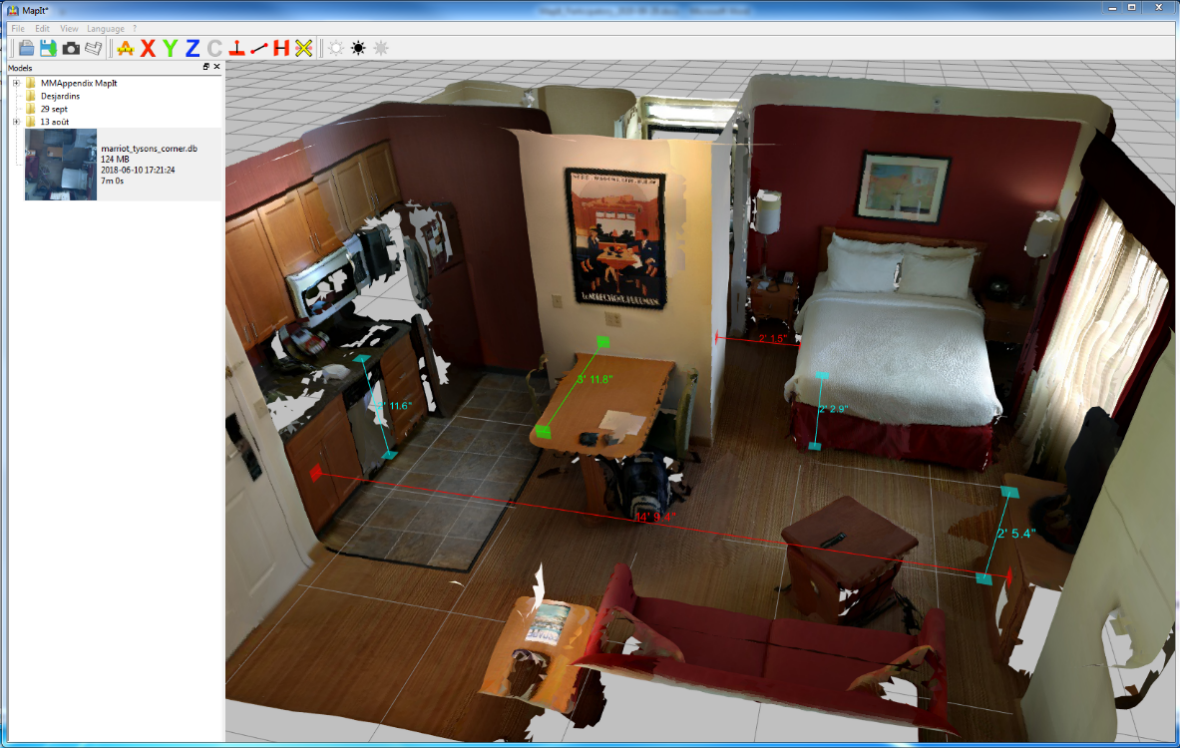
Software interface (V 0.4.2) with custom measurements taken at different locations directly from the 3D model.

The following sections describe the methods starting with Real-Time Appearance-Based Mapping (RTAB-Map; Figure 2) and a virtual repository (Figure 3), leading to an app’s visualization interface (version 0.4.1; Figure 4) and a software interface (version 0.4.2; Figure 5). An overview of the scanning process is available in video format [27,28].

### Stakeholders

#### Exploration

An informal team of 4 OTs (having 7, 15, 20, and 36 years of experiences as clinicians, 3 of them having become researchers) and 2 engineers (1 researcher and 1 postdoctoral fellow) was spontaneously formed at a scientific meeting where the idea sprouted (ie, to develop a convenient and consumer-available technology to map the home environment in 3D). While they did not have a predefined goal of addressing OTs’ home visit challenges prior to this meeting, they envisioned to explore a solution when they saw a demonstration of RTAB-Map [29,30], an existing technology allowing robots to construct a map of the environment and localize their position for autonomous navigation.

#### Pretest

At the pretest phase, an additional clinical OT and a research professional were hired to join the team. Both women had complementary expertise. The former had 25 years of experience practicing and teaching the home adaptation process, whereas the latter had mechanical engineering and cognitive ergonomics training with 4 years of experience in participatory design as a research assistant. They were supported by 2 students (1 doctoral student with a master’s in design and 1 student with a bachelor’s in psychology backgrounds), contributing to the first and second testing rounds.

#### First Testing Round

Prototyping involved additional clinical OTs during the first testing round (not part of the design team). As it was assumed that all OTs had the right to be involved in the creation of MapIt (democracy in cocreation being one of the guiding principles of participatory design [31]), a systematic sampling strategy was established with the regulatory board of OTs in Quebec. Members (N_2018_=5464 [32]) received an advertisement by email to participate in the design if they had (1) indicated a professional address in the health region where the study was conducted in 2018; (2) provided an electronic address, and (3) agreed to make their names available for research (≈80% of members). Therefore, 251 members of Quebec’s regulatory board of OTs were solicited (4.6%; personal communication with the board’s general secretary).

#### Second Testing Round

A purposeful sample was retained for the second testing round with OTs not previously involved. OTs were asked to invite their patients and patients’ relatives to provide input. Notably, when OTs could not visit the patient’s home and scan the desired rooms, patients or relatives could be referred to the designer. The designer could then organize scanning at the patient’s home with them or a relative and bring the 3D model back to their OT. In these circumstances patients or relatives were considered stakeholders.

### Tools and Techniques

#### Overview

Tools and techniques were iteratively selected to reach the study’s ultimate goal of improving the health and well-being of people engaged in a home adaptation process, by providing an acceptable technology to support this process. To reach this goal, design tools and techniques aimed to capture explicit, observable, tacit, and latent needs and knowledge (ie, what people say, do, make, test, and dream) [25,33]. The co-principal investigators (an OT and an engineer) favored a consensual leadership, promoting interdisciplinarity and encouraging creativity and mutual learning throughout the design process moving from an existing technology (RTAB-Map for robots) to one that facilitates a home adaptation process (MapIt for OTs). To gather information from the stakeholders and their context, feedback was collected throughout, allowing to continuously include their perspectives in the design process.

The different tools and techniques, the purpose they served, and also the stakeholders who created, participated in, or benefited from each tool or technique are presented in chronological order of the study’s 4 phases.

#### Exploration

MapIt is based on the RTAB-Map technology designed to help autonomous robots navigate wide indoor spaces occupied by dynamic and unstructured elements. It allows robots to construct a map of the environment and localize their position, a problem known as SLAM (Simultaneous Localization and Mapping) [29]. One of SLAM’s key attributes is the capacity to recognize an already-visited location. This is known as loop-closure detection and helps to correct errors in the map generated by sensor inaccuracy. This detection happens in real time thanks to a memory management system which limits the number of areas to compare [30]. RTAB-Map is distributed as open-source software [34]. It is included in the ROS (Robot Operating System) distribution [35], and is largely used in the robotics community.

*Demonstrations* were occasions where the RTAB-Map technology and the virtual repository were shown by the software developer to the members of the design team who had the opportunity to share impressions. The technology used to support RTAB-Map was the *Project Tango tablet* (Google) while the virtual repository was made with *Sketchfab*. Formal and informal gatherings of the design team named *workshops* took place. They were places to express creativity freely, mutually probing and answering questions. During this phase, the workshops served to envision and create a first prototype of MapIt.

#### Pretest

Overall, *prototypes* were meant to explore and imagine [26] using MapIt as a new tool for people engaged in a home adaptation process. The first prototype used during the pretest was version 0.1.0 of the MapIt smartphone app made for the *ASUS Zenfone AR*. In this phase, *demonstrations* related to version 0.1.0 of the MapIt app and were done by the software developer to the rest of the design team. *Usability testing* [23] during the pretest aimed to resolve main usability issues before testing with other stakeholders. It involved an OT researcher and a clinical OT, both members of the design team, who were thinking out loud [36] while using the prototype. During this test, *field notes* were taken by another member of the design team to document usability comments as well as interactions of the user with the app (eg, unnoticed errors, difficulties). Afterward, the clinician received an ASUS Zenfone AR with MapIt installed for a trial period of 60 working days to further test the first prototype in a clinical context. The clinician kept a *diary* to document information regarding the use of the app. Open-ended introduction and follow-up *interviews* in person or by phone between the clinician and another member of the design team allowed the collection of data regarding usability as well as barriers and facilitators to MapIt’s use in a clinical context. To keep track of ideas and observations, *logbooks* were kept by members of the design team while conducting the pretest and modifying the technology. In this phase, *workshops* served to discuss and improve the app prototype as well as establish a protocol for testing with other stakeholders.

#### First and Second Testing Rounds

Iterations of the MapIt *prototype* were made during the testing rounds. Versions 0.2.0-0.4.2 of the MapIt app (made for the *ASUS Zenfone AR*) and versions 0.3.0-0.4.2 of the software (made for *Mac OS X* and *Windows*) were developed. *Demonstrations* were related to each specific version of MapIt and were first done by the software developer to the rest of the design team. Before testing, a demonstration was done by the member of the design team conducting the test (clinical OT or research professional) to the participant (clinical OTs and relatives of their patients; not a part of the design team). This demonstration allowed to share impressions, explore learnability of the prototype, and prepare for testing. Participants also received an introduction to the technology [27] as well as to 3D scanning best practices [37] in a *video* format. After the demonstration and videos, a *usability test* [23] was officially conducted and the participant was encouraged to think out loud [36] while scanning a room with the MapIt app. While relatives only tested the app, OTs also tested the software because it was added at their request, helping them take measurements and better visualize the space. While usability tests were recorded and transcribed in the first testing round, *field notes*, taken by the person conducting the test, were added in the second testing round to accelerate the analysis process. *Questionnaires* were used to gather information from the participants and their context to better understand their perspective.

Participants were characterized using questions about their use of technology and sociodemographics. In the second testing round, the Post-Study System Usability Questionnaire (PSSUQ) [38] was added to measure perceived user satisfaction regarding the app and the software. Based on a Likert scale (1=Strongly disagree; 7=Strongly agree) with 19 items grouped into 3 subscales (system usefulness, information quality, and interface quality), 3 subscores and a total score have been calculated. Questionnaires were used to gather information from the participants and their context to better understand their perspective.

After the test session, clinical OTs received an ASUS Zenfone AR with MapIt installed, for a trial period of at least of 40 working days to further experiment with the prototype in a clinical context. Four ASUS Zenfone AR phones were available simultaneously. During this period, OTs were given a *diary* template (Multimedia Appendix 1) to document information regarding their use of the MapIt app and software.

*Interviews* were conducted by a member of the design team to have a more in-depth understanding of the OTs’ perspective. In-person semistructured interviews were conducted in the work settings at the beginning and at the end of a trial period as well as open-ended follow-up phone interviews. They were audio recorded and transcribed and the interview guides were iteratively modified (Multimedia Appendix 2).

*Workshops* were conducted with design team members to discuss and improve the app prototype following test results. During these workshops, emerging themes and conclusions were compiled and reviewed to improve the prototype. To keep track of ideas and observations, *logbooks* were kept by members of the design team while conducting tests, coding interviews, and modifying the technology. A *website* was created to access installation links, file prototype modifications, and provide access to information about the use of the app and software [39]. It was accessed by the design team members as well as by the OT participants and modified by the software developer.

### Data Analysis

Stakeholders’ characteristics (first and second rounds of testing) or satisfaction from the PSSUQ (second round of testing) was analyzed with mean and standard deviation (continuous variables) or frequency and percentage (categorical variables). To appreciate acceptability of the MapIt app and software, in the pretest and both rounds of testing, 2 or 3 members of the design team used deductive data thematic condensation with the acceptability theoretical framework [22,23] to analyze logbooks, field notes, interview transcripts, and diary texts (both available on the first and second testing rounds only). This data thematic condensation was done in Microsoft Excel, Microsoft Word, and N-Vivo 10 (QSR International). After each iteration, emerging themes were validated with stakeholders during workshops. Improvements to the prototype were made based on those themes. These modifications were then tested and evaluated in the next iteration as suggested by the user-centered iterative design process for eHealth [24]. Triangulation was achieved by drafting and editing the manuscript with coauthors.

### Research Ethics

The research protocol established for testing rounds was submitted for ethics approval, prior to involving clinicians, patients, or relatives who were not part of the research team. The study was approved by the Ethics review board of the *Centres intégrés [universitaires] de santé et services sociaux (CI[U]SSS) de l’Estrie-Centre hospitalier universitaire de Sherbrooke (CHUS)* (#2019-2827).

## Results

### Stakeholders

A total of 24 stakeholders contributed to designing MapIt, 10 being part of the team described above. The other 14 were either clinicians or relatives testing MapIt in a clinical context during first or second testing rounds.

Four OTs working in homecare settings responded to the advertisement sent by the regulatory board and were recruited (participation rate: 4/251, 1.6%) for the first testing round. Because they were all working in similar clinical settings (ie, homecare), to multiply perspectives in the second testing round, an OTs’ clinical supervisor in a geriatric health center organized a short in-person presentation of the project by the coprincipal investigator. After this, 3 OTs working in inpatient care and 2 OTs working in the day hospital signified their interest to participate by email and were recruited for the second testing round. Overall, those 8 females and 1 male were aged between 25 and 41 (mean 34 [SD 4.8] years) and had between 2 and 19 years (mean 12 [SD 5.1]) of clinical experience in occupational therapy. Seven hold a bachelor’s degree while the 2 others had completed graduate studies. Every OT owned and used a smartphone, had internet at home, and used it every day.

The relatives who provided feedback in the second testing round were 4 males and 1 female aged between 54 and 73 (mean 67 [SD 9.4] years). They had either a high-school (n=1), professional (n=2), or graduate (n=2) diploma. All of them had used and owned information and communication technology devices but only 3 of the relatives had used a smartphone. Two of the consenting patients, 1 male and 1 female, were present in their home on the day of the scan but chose not to use MapIt due to unfamiliarity with technology. The designer did the scans herself and no questionnaire was submitted to the patients.

### MapIt’s Design

As shown in Figure 1, designing of the MapIt technology involved 10 complete and 1 incomplete (app version 0.4.2 not tested) iterations. More specifically, regarding usability issues, an estimated 100 were identified and around 80 were addressed in different versions of the app and software prototypes. Examples of changes made to the prototypes between iterations to address these issues are presented in Multimedia Appendices 3 and 4. The general approach to the different iterations is explained in the following sections.

### Exploration

#### RTAB-Map

During the demonstration of RTAB-Map, OTs practicing in clinical and research fields had the opportunity to observe the construction of a map on a Project Tango tablet operated by an engineer. OTs gathered informally and suggested mapping a bathroom in the hotel to explore the possibility of using such a technology to promote occupational engagement of individuals facing architectural barriers in their home. Results were judged sufficiently promising (Figure 2).

#### Virtual Repository

A virtual repository (Figure 3) was created to assess the feasibility of conducting a participatory design study. The designer of RTAB-Map used a Project Tango tablet to create a repository of 10 bathroom scans and 3D models (RTAB-Map preliminary version on Tango tablet [40]; MapIt version 0.1.0 [28]). These models could be downloaded and examined using, for instance, MeshLab [41] or online on Sketchfab [42]. Certain types of lighting (see HouseB for example [28,40]) create significant increased camera exposure time, resulting in very bright images (almost white) and colorless patches in the 3D model. Nonetheless, it was confirmed that 3D representations of a person’s home environment could be generated. The repository supported a research grant application to move ahead.

### Pretest: Smartphone App Version 0.1.0

During the pretest, the feasibility of a smartphone app was explored. A mobile app was designed for the ASUS Zenfone AR smartphone equipped with a motion sensor and a depth perceiving camera.

During their first test with MapIt, both OTs from the design team chose to scan bathrooms. Subsequently, the clinical OT received an ASUS Zenfone AR with the app. She took 3 different bathroom scans of and took part in 2 interviews with another member of the design team.

### First and Second Testing Rounds: Smartphone App and Software Versions 0.2.0-0.4.2

During the first testing round, homecare OTs took a scan of their patient’s home on 47 occasions (35 bathrooms, 6 bedrooms, 1 dining room, 1 trailer home, 3 exterior accesses, 1 indoor staircase). During the second testing round, OTs working in inpatient care and in the day hospital did a scan of a bathroom or kitchen in their workplace in order to test the technology. Two of them also scanned bathrooms and bedrooms in their home to further explore the technology. Because OTs in the second testing round could not visit patients at home, they gave a consenting patient’s contact number to the designer who organized the scans in each patient’s home on 7 occasions. Five of these patients were hospitalized and, in each case, one of their relatives agreed to scan with MapIt (scans done by relatives: 5 bathrooms and 1 bedroom). The mean time taken by stakeholders to scan 1 room is 3 minutes and 41 seconds (range 1 minute and 6 seconds to 10 minutes and 13 seconds; SD 1 minute and 53 seconds).

In the first testing round with 4 homecare OTs, 12 semistructured interviews (mean 36 minutes; range 11-61 minutes [SD 15 minutes]) and 12 open-ended follow-up interviews (mean 12 minutes; range 3-23 minutes [SD 7 minutes]) were conducted. In the second testing round with 5 OTs working in a geriatric health center, 5 semistructured interviews (mean 37 minutes; range 27-41 minutes [SD 6 minutes]) and 15 open-ended follow-up interviews (mean 9 minutes; range 3-21 minutes [SD 5 minutes]) were carried out. The first testing round involved more recorded interviews because each usability test was included in the first interview, whereas only field notes were taken during usability tests in the second testing round. This decision was taken by the team as a way to accelerate detection of usability issues.

Including the first and second testing rounds, 14 usability tests were done either in an institution (OTs; n=9) or in a home (relatives; n=5). A total of 8 workshops happened either through formal team meeting (n=5) or scientific gathering in conferences (n=3), notably to ensure a constant focus on the context and stakeholders’ needs. For instance, in response to OTs’ comments, a computer software was designed to allow taking custom measurements on the scan and better viewing of the image. Usability tests and interviews therefore included the software from the moment the first version was released (December 9, 2018) to the end of testing (October 21, 2019).

### MapIt’s Acceptability

**Figure 6:**
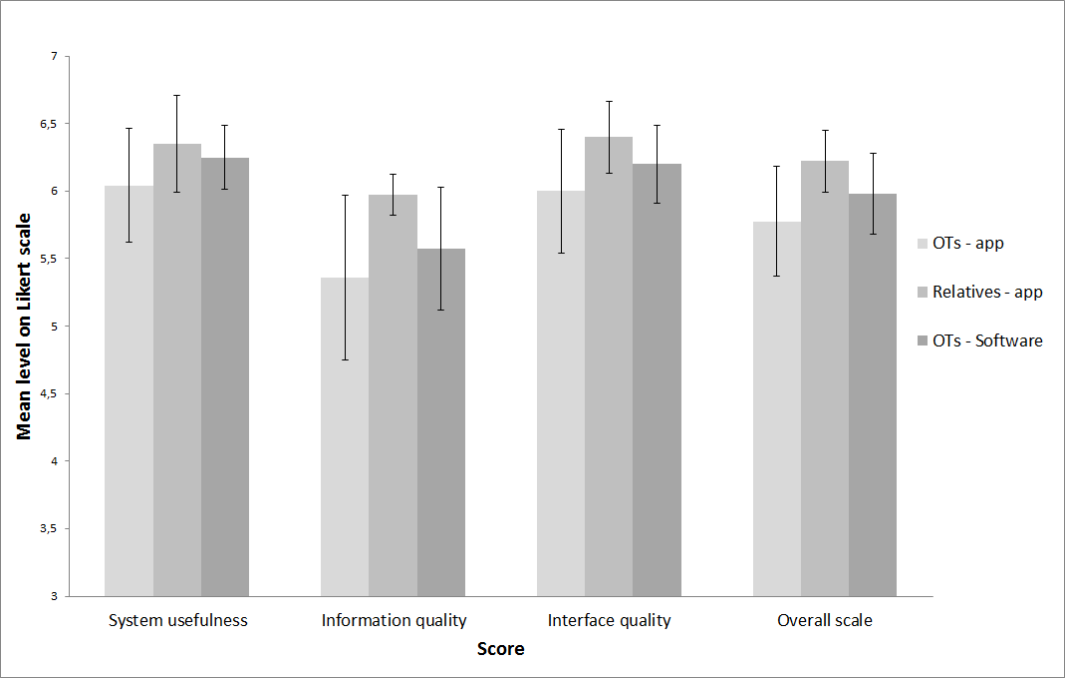
PSSUQ results in the second testing round (mean overall and subscale scores); error bars show standard error.

Stakeholders were generally satisfied with MapIt. Figure 6 presents the mean PSSUQ scores for the OTs’ and the relatives’ satisfaction regarding the app and the OTs’ satisfaction regarding the software during the second testing round. OTs and relatives “agree” with the app and the software’s system usefulness and interface quality. Information quality has lower mean scores while still being above “somewhat agree.” Stakeholders mentioned that information quality scores would have been lower had the interviewer not have helped them when they encountered a difficulty.

System acceptability, as per the Nielsen model, documented by deductively integrating the data is presented in Multimedia Appendix 5. Four identified themes (ie, utility, usability, accessibility, and scan rendering) and 14 subthemes, accompanied by their respective definition, are presented. Some citations extracted from field notes, interview transcripts, logbooks, and diaries are also presented to help understand MapIt’s acceptability as perceived by stakeholders.

MapIt’s perceived *utility* is to offer a more global view and better understanding of the patient’s environment, to take measurements, facilitate recommendations, and give a visual support to explain those recommendations. Future utilities suggested were to be able to test different home adaptations by modifying the scan, import assistive device models in the scan, and create visual reports.

The main *usability* issues outlined by stakeholders were ease of use, learnability, and efficiency. The app was easy to use by a person who is familiar with smartphones but software installation needed to be simplified in future versions. The software was less intuitive than the app and it was harder to remember how to use it when trials were spaced out. The technology has been deemed efficient and the need for efficiency was underlined.

Certain *accessibility* considerations arose when talking about the implementation of MapIt in the clinical context. For instance, some patients may feel uneasy with the fact that an image of their home is available to health professionals; 2 older adults even refused scanning in their home. When the patient gives authorization but the OT cannot visit his or her home, as was the case with OTs working in the geriatric health center, someone else must be available to scan. In this instance, the scan could be done by the patient, relatives, a government employee, or a health care professional. However, the scanning process would currently not be accessible directly to persons with major mobility or cognitive impairments. Their home would have to be scanned by someone else. Furthermore, stakeholders agreed that they would be more prone to use the technology if its access was facilitated in multiple ways: access for many stakeholders (OTs and relatives), proximity, and availability. Stakeholders also underlined the need for technical support. Comments on the cost of the technology stated that it must be kept low and, ideally, not be borne by individuals.

Stakeholders commented on the *scan rendering*. They noticed that the technology performed less well when placed in front of reflecting, dark, or single-colored surfaces. It must also be used at a certain distance from the objects scanned, which made it harder to use in small rooms. Indeed, it must be noted that image quality can be compromised by the presence of certain types of surfaces or proximity of an object. Stakeholders commented on the image quality with varying appreciation, some thinking it was good, others not. Indeed, as one familiarizes with the use of the app, the image quality of the scans produced improves. They were also concerned by the validity of measurements given by MapIt and some OTs compared them with measurements taken using a measuring tape. They argued for measurement precision, as it directly affects recommendations.

## Discussion

### MapIt Acceptability

The user-centered design of MapIt within a participatory approach aimed to maximize its acceptability for a home adaptation process, enabling individuals with disability to safely engage in their occupations. MapIt is a relatively easy-to-use mobile app available on a smartphone equipped with a motion sensor and a depth perceiving camera to scan a room and produce its 3D representation in less than 5 minutes. Resulting 3D models can be visualized on a computer software facilitating the measurement of architectural elements. OTs and the relatives of individuals living with disabilities found that MapIt is useful because it provides a global view and supports mutual understanding of a person’s environment. To initiate and maintain adoption of MapIt in the clinical context, accessibility elements have to be considered such as who will be scanning and providing technical support, all at affordable cost for an organization. Besides residual usability issues, scan rendering is a concern.

Overall, the goal is met as MapIt produces a 3D representation of person’s real home simply and rapidly. MapIt was evaluated positively as an acceptable solution, which is a crucial determinant in technology adoption according to common theories [22,23,43,44]. Compatibility has been identified previously as influencing the OT’s intention to use a technology [45]. In their study, Schaper and Pervan [45] defined compatibility as the degree to which an innovation is perceived as being consistent with the existing practices, values, needs, and experiences of the health care professional.

For instance, relying on a mobile device without a complex setup, MapIt allowed OTs as well as relatives of their patients with minimal training and supervision to scan a room in a house. It even allowed taking measurements of architectural elements remotely. It provides a 3D representation from images taken in real-world settings, one of MapIt’s key advantages, in addition to its ease of use. As wished by stakeholders, MapIt uses local processing to ensure the confidentiality mandatory in numerous eHealth interventions.

It is important to note that stakeholders were puzzled about evaluating an acceptable cost for MapIt. The approaches to be used for knowledge transfer and commercialization remain to be explored. Building a business model is a critical step to steer the adoption process of eHealth technologies [46]. It should be done keeping in mind that MapIt is currently dependent on 2 specific phones having Google Tango technology; all tests were conducted using the Asus Zenfone AR and Lenovo Phab2 Pro. However, MapIt could be ported to new Android phones equipped with a time-of-flight camera (eg, Huawei P30 Pro, Samsung Note10+). In the iOS ecosystem, the latest iPad Pro has the LiDAR technology (like a time-of-flight camera) required for MapIt, a technology which is also integrated into the new iPhone 12 Pro [47]. While MapIt has been designed for Android, porting it to iOS is now possible.

Feasible improvements are certainly good targets to allow jointly exploring home adaptations with MapIt. First, OTs would like MapIt to scale up to scanning the whole house to visualize rooms’ localization and design in relation to one another. Second, the tools for measuring architectural elements within MapIt have raised concerns about their ability to be trusted as much as the measuring tape. According to Kim and colleagues [15], having studied virtual reality as a substitution for a home visit, accuracy remains a critical concern for home modification specialists. Reliability and validity of measurements taken by a person on MapIt scans should be investigated. Third, adding shapes such as squares or circles to mimic adding a wheelchair in a bathroom or visualize a turning radius is relatively simple to do. Finally, further creative work should certainly aim to minimize residual usability issues such as difficulties inherent to the technology (eg, sensitivity to the type of surface, lighting, distance from object scanned, loop closure), which could be mitigated within the limits of the affordable and convenient technology chosen.

### Reflection About Theory

Designing an eHealth technology such as MapIt with potential users and evaluating it in a clinical context allowed to consider its acceptability from the start, as suggested by the user-centered design [24]. However, contrary to participatory design principles, the study did not truly start at the fuzzy front-end of a co-designing process [48], exploring in detail the unmet needs of people [31] involved in improving the health and well-being through home adaptations. Indeed, to determine what was to be designed or not, a participatory design process was steered toward the team members’ a priori, relying on their past research, technical, and clinical experiences. More ambiguity at the start might have led to other different solutions, whether technological or not.

Nevertheless, all study phases involved potential users, following key guiding participatory design principles such as democracy, mutual learning, and collective creativity [31]. A more targeted approach allowed us to focus on a tangible solution to increase potential success with academic grants. This approach combined a substantial number of tools and techniques into a coherent design process. Applicable results were pursued to move relatively quickly and test a solution in a clinical context.

Yet, welcoming the expression of all needs during designing (ie, explicit, observable, tacit and latent) and looking for what people say, do, make, test, and dream [25,33] poses the challenge of prioritizing the (endless) possibilities during a (non-eternal) research study. For example, the request of OTs to be able in the future to delete, add, or move architectural elements (eg, cabinets in the bathroom) on a scan must be balanced with the cost of such a technological development, in a context where other available technologies already address this need [16-21]. Interdisciplinary consensual coleadership has probably contributed to dealing efficiently with inherent tensions and encouraged open creative thinking, while focusing on getting to a clinical hands-on solution.

### Study Limitations

One study limitation results from the fact that stakeholders were not left to fend for themselves if a problem occurred during usability testing. This decision was made because the MapIt technology is not similar to any commonly used app or software. Although errors were recorded, stakeholders could ask questions and receive help from the interviewer. This did not allow to collect usability metrics relating to effectiveness (level of completion of task) and allowed limited metrics on efficiency (time to complete task) [23], but it did allow to collect impressions throughout the whole scanning and viewing tasks. In terms of usability, qualitative data were judged to be more important due to the stage of the technology development and the size of the sample. Future studies should involve testing without support of an expert.

Another limitation comes from sample size. While it includes different user groups, the sample is small which limits generalization. However, 2 rounds of testing were done, and the prototype was improved in an iterative fashion: the sample is sufficient to dig below surface value insight into usability issues [49,50]. Other stakeholders involved in the home adaptation process, such as paying authorities, builders, and interdisciplinary health care team members [51], were not solicited. Broader perspectives of stakeholders might have enhanced the participatory design even more. Still, a design team comprising OTs, engineers, clinicians, and students provided ongoing input from the start of the study, as suggested by participatory design, and input from lay OTs, older adults, and their relatives was added during testing rounds, which is coherent with a user-centered process for the development of an eHealth technology [24].

### Conclusions

MapIt is an eHealth solution developed through a user-centered and participatory process perceived by stakeholders as an acceptable technology to jointly explore home adaptations overcoming environmental barriers to enhance the independence of individuals with disabilities. This mobile app mapping a room to produce a 3D representation of a “real” home with a smartphone is useful because it was relatively easy to use, contributing to OTs work by providing a global view and supporting mutual understanding of a person’s environment. As with other eHealth interventions, accessibility considerations must be addressed to support adoption in the clinical context while MapIt’s usability and scan rendering will be improved.

## Appendix

Multimedia Appendix 1 Documentation of MapIt's use.

Multimedia Appendix 2 Interview guides.

Multimedia Appendix 3 Smartphone app prototype modifications (examples).

Multimedia Appendix 4 Computer software prototype modifications (examples).

Multimedia Appendix 5 Acceptability of MapIt and its contribution to the work of OTs.

## Abbreviations

OT: occupational therapist
PSSUQ: Post-Study System Usability Questionnaire
ROS: Robot Operating System
RTAB-Map: Real-Time Appearance-Based Mapping
SLAM: Simultaneous Localization and Mapping

## References

[1] Fried TR, Bradley EH, Williams CS, Tinetti ME (2001). Functional disability and health care expenditures for older persons. Arch Intern Med.

[2] Carnemolla P, Bridge C (2016). Accessible housing and health-related quality of life: Measurements of wellbeing outcomes following home modifications. ArchNet-IJAR.

[3] Stark S, Keglovits M, Arbesman M, Lieberman D (2017). Effect of Home Modification Interventions on the Participation of Community-Dwelling Adults With Health Conditions: A Systematic Review. Am J Occup Ther.

[4] Morgan R, DiZazzo-Miller R (2018). The Occupation-Based Intervention of Bathing: Cases in Home Health Care. Occup Ther Health Care.

[5] Lannin NA, Clemson L, McCluskey A, Lin CC, Cameron ID, Barras S (2007). Feasibility and results of a randomised pilot-study of pre-discharge occupational therapy home visits. BMC Health Serv Res.

[6] Atwal Anita, Spiliotopoulou Georgia, Stradden Jennifer, Fellows Victoria, Anako Emma, Robinson Lisa, McIntyre Anne (2014). Factors influencing occupational therapy home visit practice: a qualitative study. Scand J Occup Ther.

[7] Harris S, James E, Snow P (2008). Predischarge occupational therapy home assessment visits: towards an evidence base. Aust Occup Ther J.

[8] Sim S, Barr CJ, George S (2015). Comparison of equipment prescriptions in the toilet/bathroom by occupational therapists using home visits and digital photos, for patients in rehabilitation. Aust Occup Ther J.

[9] Daniel H, Oesch P, Stuck AE, Born S, Bachmann S, Schoenenberger AW (2013). Evaluation of a novel photography-based home assessment protocol for identification of environmental risk factors for falls in elderly persons. Swiss Med Wkly.

[10] Romero S, Lee MJ, Simic I, Levy C, Sanford J (2018). Development and validation of a remote home safety protocol. Disabil Rehabil Assist Technol.

[11] Latulippe K, Provencher V, Boivin K, Vincent C, Guay M, Kairy D, Morales E, Pellerin M, Giroux D (2019). Using an Electronic Tablet to Assess Patients' Home Environment by Videoconferencing Prior to Hospital Discharge: Protocol for a Mixed-Methods Feasibility and Comparative Study. JMIR Res Protoc.

[12] Latulippe K (2020). La visioconférence mobile pour évaluer le domicile : une revue rapide. Revue Francophone de Recherche en Ergothérapie.

[13] Money AG, Atwal A, Young KL, Day Y, Wilson L, Money KG (2015). Using the Technology Acceptance Model to explore community dwelling older adults' perceptions of a 3D interior design application to facilitate pre-discharge home adaptations. BMC Med Inform Decis Mak.

[14] Atwal A, Money A, Harvey M (2014). Occupational therapists' views on using a virtual reality interior design application within the pre-discharge home visit process. J Med Internet Res.

[15] Kim J, Brienza DM, Lynch RD, Cooper RA, Boninger ML (2008). Effectiveness evaluation of a remote accessibility assessment system for wheelchair users using virtualized reality. Arch Phys Med Rehabil.

[16] Idapt planner http://www.idapt-planning.co.uk/room_layout_planner/index.php.

[17] OT Draw http://otdraw.com/.

[18] SketchUp https://www.sketchup.com/.

[19] SweetHome 3D http://www.sweethome3d.com/.

[20] Matterport https://matterport.com/.

[21] CANVAS https://canvas.io.

[22] Nielsen J (1993). Usability engineering.

[23] International Organization for Standardization (2018). ISO 9241-11:2018 Ergonomics of human-system interaction. Part 11: Usability: Definitions and concepts.

[24] De VDA, Myers BA, Mc CKR, Dunbar-Jacob J, Hawkins RP, Begey A, Dew MA (2009). User-centered design and interactive health technologies for patients. Comput Inform Nurs.

[25] Dell'Era C, Landoni P (2014). Living Lab: A Methodology between User-Centred Design and Participatory Design. Creativity and Innovation Management.

[26] Sanders EB, Stappers PJ (2014). Probes, toolkits and prototypes: three approaches to making in codesigning. CoDesign.

[27] Introlab MapIt(EN).

[28] RTAB-Map Tango: Bathrooms HD [MapIt version 0.1.0].

[29] Labbé M, Michaud F (2017). Long-term online multi-session graph-based SPLAM with memory management. Auton Robot.

[30] Labbe M, Michaud F (2013). Appearance-Based Loop Closure Detection for Online Large-Scale and Long-Term Operation. IEEE Trans. Robot.

[31] Vandekerckhove P, de Mul M, Bramer WM, de Bont AA (2020). Generative Participatory Design Methodology to Develop Electronic Health Interventions: Systematic Literature Review. J Med Internet Res.

[32] Ordre des ergothérapeutes du Québec, Rapport annuel 2017-2018 (2018). https://www.oeq.org/a-propos-de-l-ordre/salle-de-presse/actualites/72-le-rapport-annuel-2017-2018-est-maintenant-disponible.html?page=9.

[33] Sanders E (2002). , From User-Centered to Participatory Design Approaches, in DesignSocial Sciences: Making Connections, T. Francis, Editor: London.. ISBN.

[34] RTAB-Map.

[35] RTAB-Map. ROS.

[36] Nielsen J (2012). Thinking aloud: The #1 usability tool.

[37] Canvas 3D Scanning Best Practices,. Occupital HQ.

[38] Lewis JR (2002). Psychometric Evaluation of the PSSUQ Using Data from Five Years of Usability Studies. International Journal of Human-Computer Interaction.

[39] Introlab MapIt.

[40] RTAB-Map preliminary version on Tango tablet.

[41] MeshLab. www.meshlab.net.

[42] Sketchfab www.sketchfab.com.

[43] Davis FD (1989). Perceived Usefulness, Perceived Ease of Use, and User Acceptance of Information Technology. MIS Quarterly.

[44] Venkatesh, Morris, Davis, Davis (2003). User Acceptance of Information Technology: Toward a Unified View. MIS Quarterly.

[45] Schaper LK, Pervan GP (2007). ICT and OTs: a model of information and communication technology acceptance and utilisation by occupational therapists. Int J Med Inform.

[46] van Gemert-Pijnen JEWC, Nijland N, van Limburg M, Ossebaard HC, Kelders SM, Eysenbach G, Seydel ER (2011). A holistic framework to improve the uptake and impact of eHealth technologies. J Med Internet Res.

[47] Apple iPhone 12 Pro.

[48] Sanders EB, Stappers PJ (2008). Co-creation and the new landscapes of design. CoDesign.

[49] Nielsen J (2000). Why you only need to test with 5 users.

[50] Nielsen J (1993). and T. K. Landauer. A mathematical model of the finding of usability problems. in INTERACT'93 and CHI'93.

[51] Ainsworth E (2019). and D. de Jonge, An Occupational Therapist's Guide to Home Modification Practice.

