# Supplementary material for: Adapting a Person’s Home in 3D Using a Mobile App (MapIt): Participatory Design Framework Investigating the App’s Acceptability
Source: JMIR Rehabil Assist Technol. 2021 May 11;8(2):e24669. doi: 10.2196/24669 (PMC8150410; doi:10.2196/24669)
Supplement: Multimedia Appendix 1 [file rehab_v8i2e24669_app1.pdf]

participant #: ________

DOCUMENTATION OF MAPIT'S USE
(OT's Diary)

<table>
<tr><td rowspan="7">Date :
J J / M M /
A A A A</td><td rowspan="6">MapIt used (indicate « phone » or « computer »)</td><td>Number of clients</td><td></td><td>Client type(s)</td><td></td></tr>
<tr><td>Context(s)</td><td colspan="3"></td></tr>
<tr><td>Positive aspects +</td><td colspan="3"></td></tr>
<tr><td>Negative aspects -</td><td colspan="3"></td></tr>
<tr><td>Questions/Comments</td><td colspan="3"></td></tr>
<tr style="display:none"></tr>
<tr><td>MapIt not used</td><td>Reasons</td><td colspan="3"></td></tr>
<tr><td rowspan="6">Date :
J J / M M /
A A A A</td><td rowspan="5">MapIt used (indicate « phone » or « computer »)</td><td>Number of clients</td><td></td><td>Client type(s)</td><td></td></tr>
<tr><td>Context(s)</td><td colspan="3"></td></tr>
<tr><td>Positive aspects +</td><td colspan="3"></td></tr>
<tr><td>Negative aspects -</td><td colspan="3"></td></tr>
<tr><td>Questions/Comments</td><td colspan="3"></td></tr>
<tr><td>MapIt not used</td><td>Reasons</td><td colspan="3"></td></tr>
<tr><td rowspan="6">Date :
J J / M M /
A A A A</td><td rowspan="5">MapIt used (indicate « phone » or « computer »)</td><td>Number of clients</td><td></td><td>Client type(s)</td><td></td></tr>
<tr><td>Context(s)</td><td colspan="3"></td></tr>
<tr><td>Positive aspects +</td><td colspan="3"></td></tr>
<tr><td>Negative aspects -</td><td colspan="3"></td></tr>
<tr><td>Questions/Comments</td><td colspan="3"></td></tr>
<tr><td>MapIt not used</td><td>Reasons</td><td colspan="3"></td></tr>
</table>
