# Supplementary material for: Adapting a Person’s Home in 3D Using a Mobile App (MapIt): Participatory Design Framework Investigating the App’s Acceptability
Source: JMIR Rehabil Assist Technol. 2021 May 11;8(2):e24669. doi: 10.2196/24669 (PMC8150410; doi:10.2196/24669)
Supplement: Multimedia Appendix 2 [file rehab_v8i2e24669_app2.docx]

Multimedia Appendix 2. Interview guides

*[Probe]* Always add “is there anything else?” until participants has nothing more to add

1. How is your trial of MapIt going?
2. What difficulties did you encounter while using MapIt?
3. Please name some advantages that you were able to identify with MapIt?
4. (If MapIt has not yet been used) For what reasons have you not used MapIt yet?
5. Which uses of MapIt did you identify (even if not tested yet)?
6. Would you like to share something with me that we did not address?
7. How did you find this follow-up meeting?
8. Is there anything else I can do to accompany you for the remainder of you trial period with MapIt?

Sample final interview guide

1. How would you qualify your experience with MapIt?
2. How was MapIt useful in your work?

*[Probes]*

- 1. *I would like to better understand your idea, what do you mean by that?*
  2. *How did you use the scan afterwards?*
  3. *How was the scan useful in your work?*
  4. *With which colleagues did you use MapIt and in which way did you use it?*
  5. *What other potential uses could there have been for MapIt in your occupational therapy practice?*

1. Now that you have experienced the use of MapIt, how would you see it being useful to other OTs?

*[Probes]*

- 1. *In what manner?*
  2. *In what context?*
  3. *Who would be a potential user of MapIt?*
  4. *What other potential applications could MapIt have for OTs in general?*

1. What should we change in MapIt?

*[Probes]*

- 1. *What more could MapIt do for you?*
  2. *What do you find displeasing with MapIt?*

1. According to you, in which direction should we focus our efforts to ensure MapIt’s development?
2. What do you envision the future of MapIt?

*[Probes]*

- 1. *In occupational therapy?*
  2. *In other fields?*

1. What were the obstacles to MapIt’s use in your clinical practice?
2. What helped you include MapIt in your work?
3. Could you describe how you would think that MapIt could be made available?
4. Now that you have tried MapIt, how much would you be ready to pay to obtain this technology for your clinical practice?
5. What more would you like to tell me about MapIt?

*[Probe]*

- 1. Is there anything else? (until participant has nothing more to add)
