# Supplementary material for: Adapting a Person’s Home in 3D Using a Mobile App (MapIt): Participatory Design Framework Investigating the App’s Acceptability
Source: JMIR Rehabil Assist Technol. 2021 May 11;8(2):e24669. doi: 10.2196/24669 (PMC8150410; doi:10.2196/24669)
Supplement: Multimedia Appendix 3 [file rehab_v8i2e24669_app3.pdf]

Smartphone app prototype modifications (examples)

Feature before	Version(s)	Data collection	Feature after	Version
App coding only	0.1.0	*High consumption on Idle, disable Tango when we are on visualization [Logbook ML]*	App coding only	0.2.0
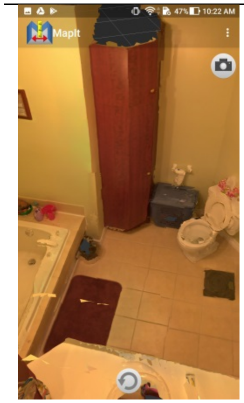 Scan visualization without measurement option on version 0.1.0	0.1.0	*Measurements would be useful to OTs [Logbook NC]*	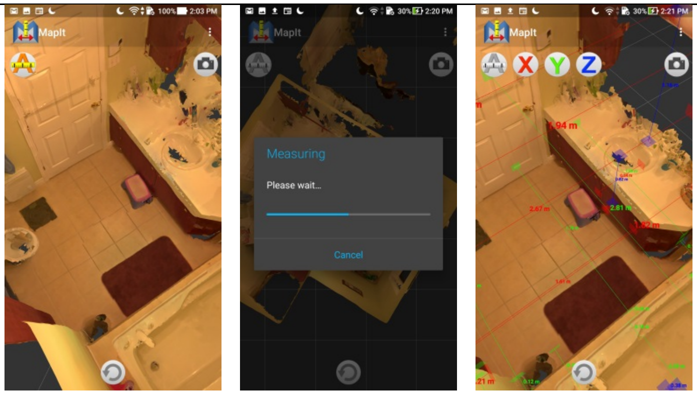 In visualization mode, version 0.2.0, press the “A” icon to activate automatic measurements. The app detects plane surfaces and uses them as start and end points of measurements.	0.2.0
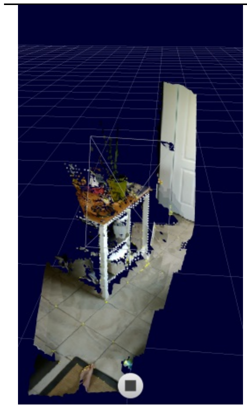 Recording screen on version 0.1.0	0.1.0	*Add flashing "REC" indication when recording [Logbook – NC]*	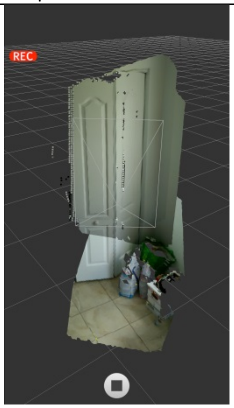 On version 0.3.0, a “Rec” flashing red indication is added to the recording screen.	0.3.0
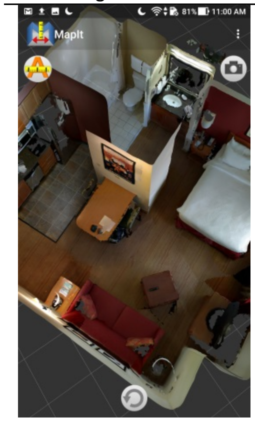 Visualization screen on version 0.2.0 (no top view)	0.1.0, 0.2.0	*If there was an option for a top view, we could see the room measurements [...] this would simplify the following work of finding solutions and adaptations. [Interview3 – P1]*[a]	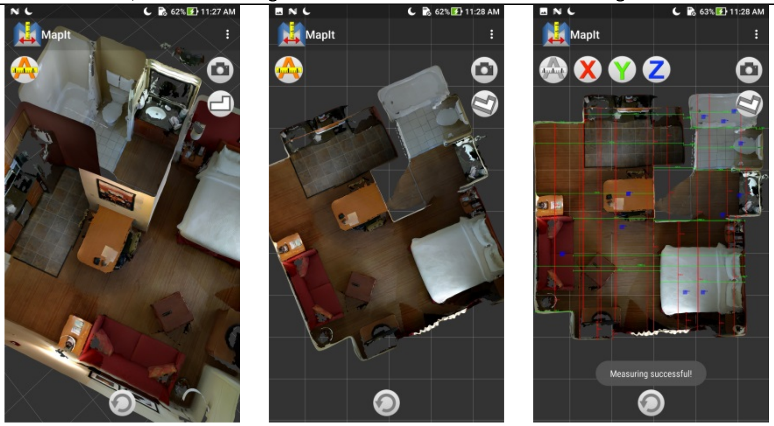 From visualization screen on version 0.3.0, press the top view icon (below the camera icon) and the “A” icon to see room measurements from a top view.	0.3.0

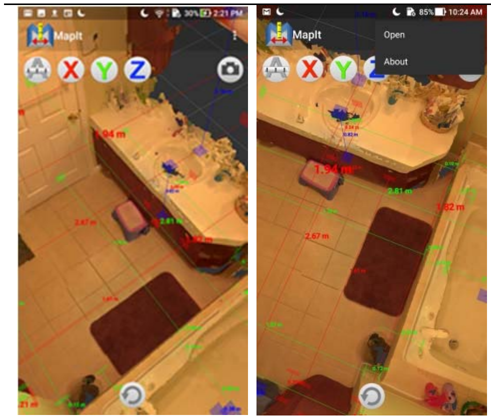<br>Automatic measurements are only presented in meters (no option in top right menu).	0.2.0	*Would it be possible to see the measurements in inches rather than in meters? When I compare, I always have to make the conversion because most of our assistive equipment is in inches in the equipment loan system. [Diary Day10-P2]*	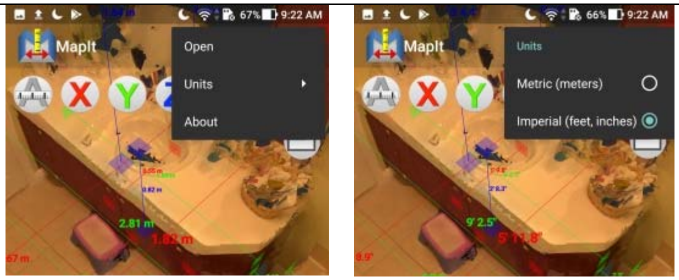<br>With automatic measurements, go to top right menu to change units.	0.3.0
Absent	0.1.0, 0.2.0	*For certain measurements, I find it's even more important, like a door opening, but it [the app] does not give this measurement. [Interview2 – P3]*<br><br>*Visualization of the environment with the measurements is challenging on the cellphone. Too many measurements for a small screen. General view insufficient [Diary Day1 – P1]*	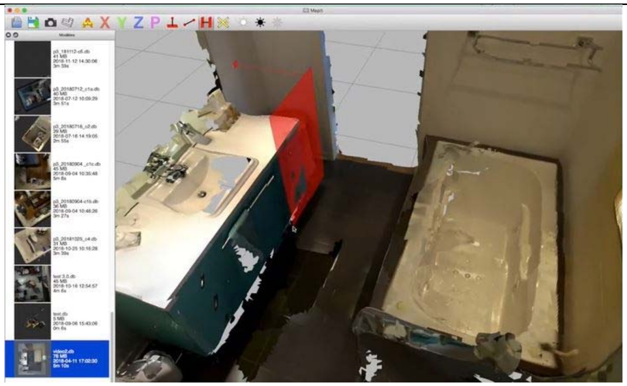<br>Custom measurements and visualization on a larger screen were made possible by the introduction of the MapIt Software.	0.3.0 - On computer (see Multimedia Appendix 4)
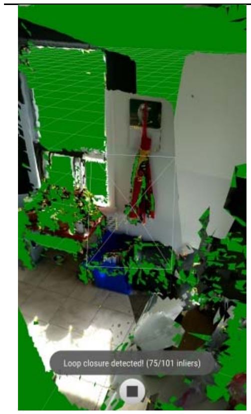<br>Loop detection on version 0.1.0	0.1.0, 0.2.0, 0.3.0	*Most participants say loop closure is hard. [Logbook – NC]*<br><br>*Color change on screen makes loop closure clear [Diary Day25 – P2]*<br><br>*Sometimes it [loop closure] does not work. It is necessary to stay still for a couple of seconds and have the right distance and angle with the cellphone. People have the intuition to move until loop closure is detected. [Logbook – NST]*	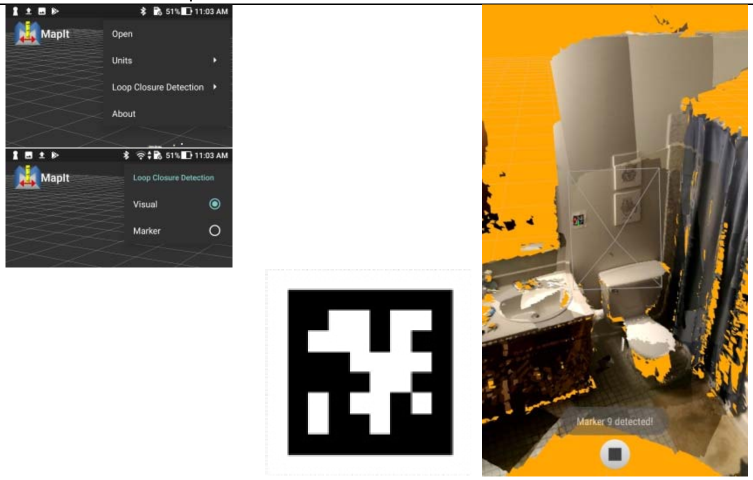<br>A Marker mode was added in version 0.4.1. To use it, under Loop Closure Detection option in menu, choose Marker and use image (similar to QR code) provided on Introlab	0.4.1

			website (https://introlab.3it.usherbrooke.ca/mediawiki-introlab/index.php/MapIt#Tags) as starting and end point of scan. Note: Detection of starting point and loop closure is immediate when using Marker mode and indicated by a yellow background and an onscreen message. This mode was not tested in this study.	
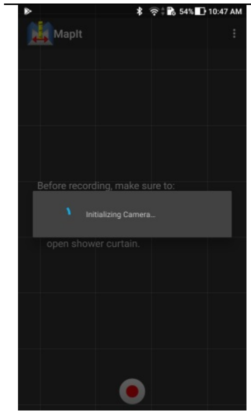<br>On all versions before 0.4.2, when opening MapIt app, the camera initializes and scanning mode is chosen by default.	0.1.0, 0.2.0, 0.3.0., 0.4.1	*Ideally, […] when we open the app, it would be nice to have a menu giving the option to open the camera or a previous scan. [Interview 4 – P3]*	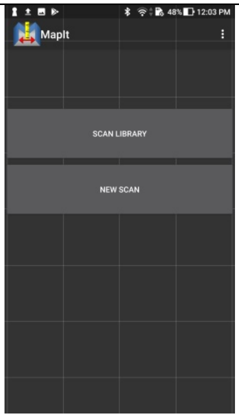 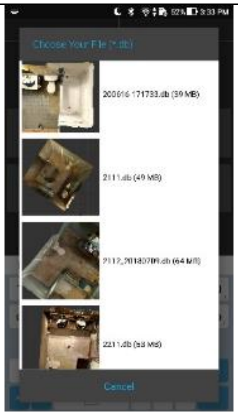<br>On version 0.4.2, when opening MapIt, a choice is given to either open the scan library (to choose a scan for viewing) or to create a new scan.	0.4.2[b]
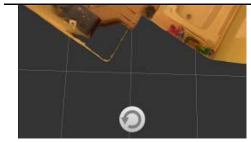<br>On all versions before 0.4.2, an icon similar to a Reset button is used to change from visualization screen to scanning mode.	0.1.0, 0.2.0, 0.3.0, 0.4.1	*Does not understand how to go back to the scanning option after visualization of a scan on the smartphone app. [Field notes – NST]*	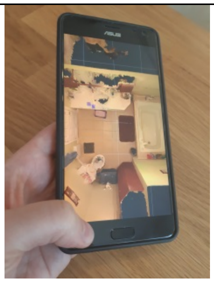 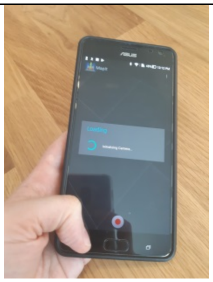 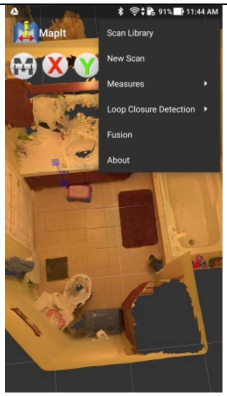<br>Version 0.4.2 uses the smartphone's "back" function to exit visualization screen and to exit app. In visualization mode, a "New scan" option in the top right menu can also be chosen.	0.4.2

Absent	0.1.0,<br>0.2.0,<br>0.3.0,<br>0.4.1	*Generate custom measurements on the map (directly on the smartphone without transferring the map to the computer) [Logbook – NC]*<br><br>*Would have liked to take measurements directly on the smartphone (does not give commonly used measurements like the toilet, the bath and the sink with the automatic function) [Field notes – NST]*	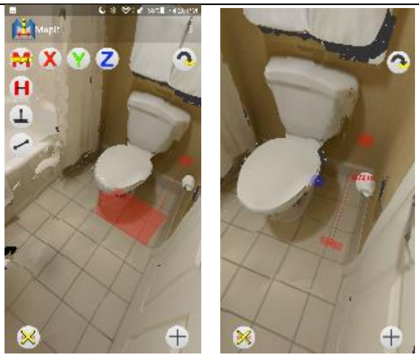<br>A virtual reality style measuring mode has been added to version 0.4.2. Watch a demonstration : https://www.youtube.com/watch?v=xr-ACgqpUlk&feature=youtu.be	0.4.2
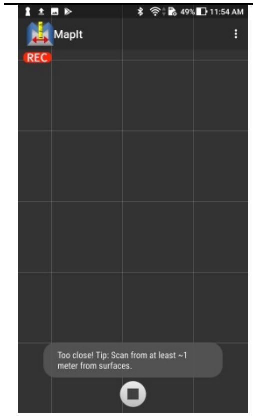<br>In all versions before 0.4.2, messages appear in grey at the bottom on the screen.	0.1.0,<br>0.2.0,<br>0.3.0,<br>0.4.1	*Messages appear too low on the phone screen, the participant is focused on the scan image and does not see the messages [Logbook – NST]*	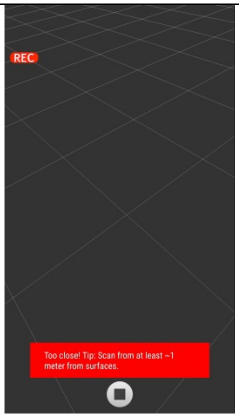<br>On version 0.4.2, messages appear in red to be more noticeable.	0.4.2

a. Comments given by OTs were either collected through interviews or field notes. When comments were collected from interview transcripts, the interview number and participant number are given. When the comments were collected through field notes, the initials of the person taking these notes are given.
b. V 0.4.2 of the app prototype was not tested in this study.
