# Supplementary material for: Adapting a Person’s Home in 3D Using a Mobile App (MapIt): Participatory Design Framework Investigating the App’s Acceptability
Source: JMIR Rehabil Assist Technol. 2021 May 11;8(2):e24669. doi: 10.2196/24669 (PMC8150410; doi:10.2196/24669)
Supplement: Multimedia Appendix 4 [file rehab_v8i2e24669_app4.pdf]

Computer software prototype modifications (examples)

Feature before	Version(s)	Data collection	Feature after	Version
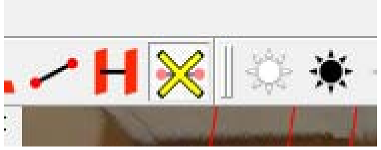	0.3.0	*Every time I want to erase a measure, I have to press the option again which is less practical [Interview4 – P1]*	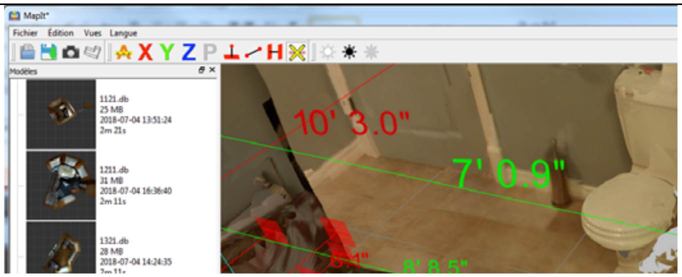 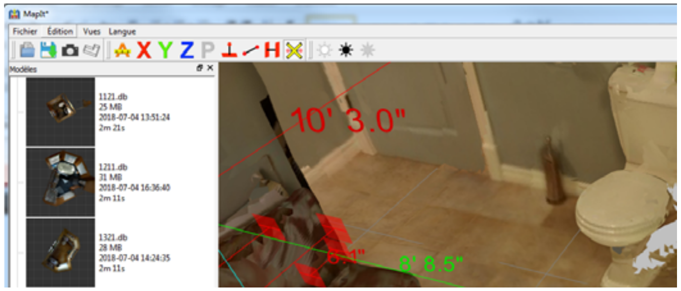 *The "Remove measurement" button stays active as long as it is not clicked a second time [Logbook – ML]*	0.4.0
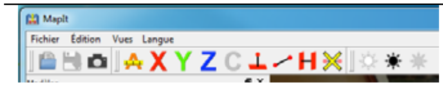 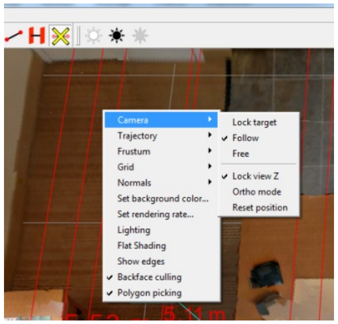	0.3.0	*Change the name "Ortho mode": it is not meaningful [Logbook – NC]*<br><br>*Since "Ortho mode" is often used, why is it not included in the tool bar on the top of the screen? [Logbook – NC]*<br><br>*In the right click menu there is a function "Camera -> Ortho mode" that I would have put in the tool bar. [Interview6 – P2]*	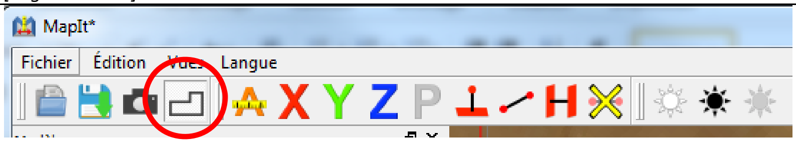 *Added a "Plan view" button in the tool bar on the top of the screen.*<br>*This replaces the Camera->Ortho mode function in the right click menu. [Logbook – ML]*	0.4.0
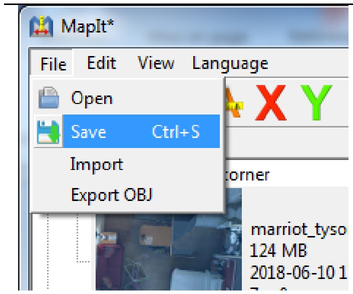	0.3.0	*Absence of the option to save as. Saving then automatically deletes the original version. [Diary Day12 – P1]*	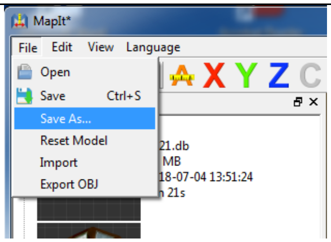 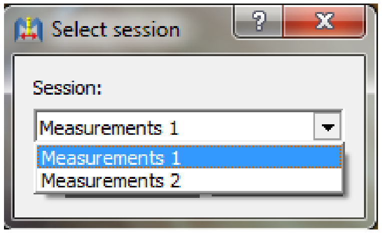 *Many measuring sessions can be saved in the same model with File -> Save As... to save measurements in a new session. When a model comprises more than one measurement*	0.4.0

			session, the desired session can be chosen from a scroll down menu in a dialog box when opening the model. [Logbook – ML]	
	0.4.0	*What does Polygon Picking mean? [Interview6 – P2]*<br>*Right click language stays in English even if French option was chosen in Language menu [Logbook – NC]*	*Correction of translation problems in dialog buttons [Logbook – ML]*	0.4.1
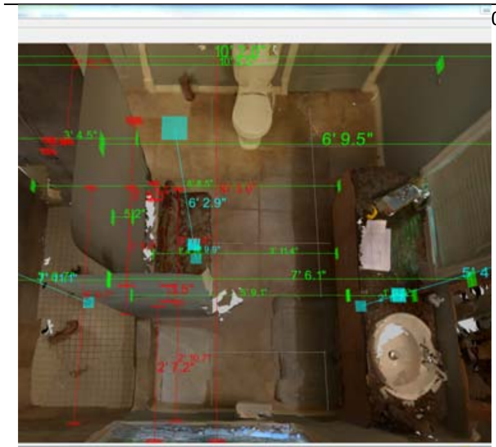	0.4.0	*The software generates too many automatic measurements [Logbook – NC]*	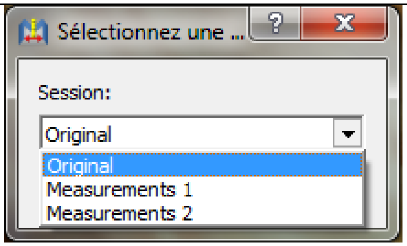<br>*Addition of an "Original" measuring session (without automatic measurements) in the dialog box [Logbook – ML]*	0.4.1
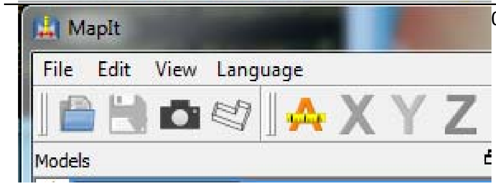	0.4.0	*Add more information to software such as version number and help button [Logbook - ML]*	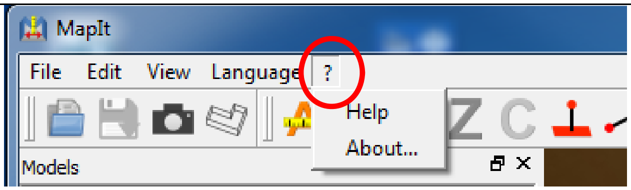<br>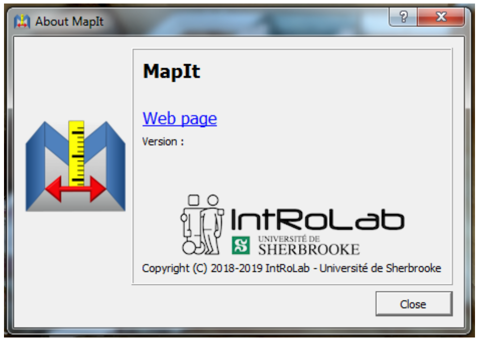<br>*Addition of a "?" menu containing "Help", redirecting to https://introlab.3it.usherbrooke.ca/mediawiki-introlab/index.php/MapIt#Utilisation_de_MapIt, and "About..." with a dialog box linking to same webpage. [Logbook – ML]* (Note: version number was added to subsequent version 0.4.2)	0.4.1
Software coding only	0.4.1	*When trying to open a scan on the software, I have a "fatal error" message and the software crashes [Logbook – NST]*	*Software coding only*<br>*Solved an incompatibility between the last software version and app version following import of a model [Logbook – ML]*	0.4.2
