# Supplementary material for: Adapting a Person’s Home in 3D Using a Mobile App (MapIt): Participatory Design Framework Investigating the App’s Acceptability
Source: JMIR Rehabil Assist Technol. 2021 May 11;8(2):e24669. doi: 10.2196/24669 (PMC8150410; doi:10.2196/24669)
Supplement: Multimedia Appendix 5 [file rehab_v8i2e24669_app5.docx]

**Acceptability of MapIt and its contribution to the work of OTs**

| **Themes** | **Definitions** | **Citations** |
| --- | --- | --- |
| **Utility** | What can and will it be used for in the stakeholder’s context |  |
| Current | Use of the technology as it is | *There has to be a certain number of inches for a bath board. It [MapIt] allowed me to assess this. [Follow-up3 – E1]*  *[…] I find MapIt is a bit more… I would say “dynamic”* *[…] I am able to relate with the third dimension of the space without necessarily needing precise measurements […] It [MapIt] allowed me to have a global view of the home. [Interview – E7]*  *Instead of having it on a small two-dimensional sketch, it’s seeing the bathroom and studying it. [Interview1 – P1]*  *There are certain recommendations, the more routine recommendations that would be easy to make with the scan. If we landed on an environment where there were no grab bars, it would have been easy for me to recommend adding them as soon as possible. [Interview2 – P1]*  *Because this patient had small understanding difficulties, when I talked to him face to face like this, it was not concrete enough. But having the image of his own shower, it was easier for him to understand the modifications I was suggesting. [Interview – E7]* |
| Future | Potential use of the technology with suggestions of added functionalities | *Having a text box to add information on the scan [Logbook – NC]*  *Generate visual reports (PDF, 3D model) for documentation and formulation of recommendations* *[Logbook – NC]*  *Being able to import assistive device models in the .db file [MapIt software file] for visualization* *[Logbook – NC]*  *Would like to be able to draw a circle to measure a turning radius (eg, wheelchair) [Logbook – NST]*  *I would find it practical to be able to select a section of the 3D capture (eg, bathroom vanity cabinets) and delete it or move it to help visualize different layout scenarios with a 3D vision. [Interview1 – P1]*  *OTs would like to see the rooms in relation to one another since certain architectural modifications affect more than one room at a time. [Logbook - MG]* |
| **Usability** | Main usability issues |  |
| Ease of use | Ease with which it can be used | *[…] even my eight year old boy scanned his room and it went well. [Follow-up1 – E7]*  *Otherwise, manipulation of the smartphone was easy enough. [Interview3 – P1]*  *Some will get it at once, but sometimes there is a brother who does it. The brother of an 80 year old person is not necessarily skilled with a smartphone. [Interview – E2]*  *At the start it’s a bit arduous [MapIt software]. I am good with computers but the icons, open this, open that, I was a bit lost there. [Interview – E5]*  *Sure the installation on a computer was quite long and complicated. [Follow-up1 – P1]* |
| Learnability | Learning necessary to use the technology | *Because regarding the software, I realised that when I spaced out its use, I always had to remember how to move the scan […], how to zoom, how to take measurements. [Interview E7]*  *Sure it was the third time I was trying [the software] so it was becoming a bit more automatic. [Follow-up2 – E2]* |
| Efficiency | Efficiency in the use of the technology | *I found this tool [app] was quite fast. [Interview2 – P4]*  *It has to be fast and efficient to be appreciated. [Interview – E5]*  *Yes, a teaching would be nice, but there is a question of efficiency […] we would have to meet once before the scan. [Interview – E2]* |
| **Accessibility** | Organisation surrounding the use in the stakeholder’s context |  |
| Capacities necessary to complete a scan | Accessibility for people with different capacities | *We have to bend [to scan] the toilet, patients with low mobility would probably have difficulty doing it by themselves. [Diary – E6]*  *But to do the scan, I don’t know, will it really be the patients? Patients for whom I find MapIt useful have too much [cognitive] damage to really do it by themselves. [Follow-up3 – E6]* |
| Patient authorisation | Obtaining the patient's authorisation to scan the home and to keep the scan | *Refusal from client. Inability to make him understand the goal of the research project. [Diary – P1]*  *Project presented to the patient: fears that someone will see the dust, the messiness, etc. so refuses to participate. [Diary – E7]*  *The fact also that […] a scan would be captured made him [the patient] more nervous. If it would have been only me going to his house, we have a trusting relationship and it would have been ok, but the fact that there would be a trace bothered him. [Follow-up1 – E7]* |
| Scan in the patient's home | Availability and collaboration of the family, caregiver or another person for the scan in the patient's home | *Yes, I would let patients or their close ones, their relatives, use it if it is super simple. […] Q: This would be a way to make it available for you? You would have it as a tool and lend it? A: Yes. [Interview – E1]*  *As a tool here, to have it available to us, I am not sure. Having someone with freed up time, who is paid by the government to do this, the I’m all in. [Interview – E5]*  *[…] so I wouldn’t give the smartphones to OTs but to others such as the social worker, the nurse […] they could scan the environment so that we could prepare and act in the most rapid and precise way. [Interview2 – P1]* |
| Technology access | Getting hold of the technology | *Yes, they would get it directly on the App Store […] versus a phone that we have to lend to the families because, once again, they would have to come here to get the phone. [Interview – E2]*  *Q: According to you, what is MapIt’s greatest future in the field of occupational therapy? A: For me it’s MapIt’s accessibility, it’s allowing the patients to use the phone by themselves. [Interview – E7]*  *So I think that if we had one phone [with MapIt] between the three of us [OTs] it would be ok. If we had to share it for the whole building, I think it would be an issue. I would have a tendency to not use it as much, a bit like the Wii console we have on the third floor. I love using it but I have to walk a long distance with the patient so I use it less. If I had it here I would use it every day. [Interview – E7]*  *[…] the more people in a team sharing the same phone, the less it is available and the less available, the less usable. [Interview2 – P1]* |
| Technical support | Need for technical support | *Q: So in your case, would there be a need for support? A: Surely, yes, because when I had questions or there were bugs I needed someone who knew the technology to help me. [Interview – E6]*  *Yes, so maybe if I hadn’t had [the designer] to assist with this, I would have encountered my first difficulty and I would have put it [MapIt app] aside. I would have been highly possible. [Interview – E7]*  *I don’t know, I imagine that there would be […] not a user’s guide but something like that that would come with it. [Follow-up2 – P2]* |
| Cost | Acceptable cost of the technology and who would pay | *I would see my workplace buying the technology with the app […] and making it available to us […] I would see it like that. It’s a work tool. [Interview – E1]*  *If we make it available to patients, I am not sure they would pay. Should it be like a subscription to a service and the patients could have a temporary password for a couple of day’s access? Would I pay? I think it could be cost-efficient. I don’t know how much. It’s a good question. It depends if it’s individual, the price, or if it is a group fee […] I don’t know.* [Interview – E6] |
| **Scan rendering** | Visual aspect of the scan |  |
| Features of the room | Features of the room being scanned influencing the scanning process and the resulting scan quality | *Good image quality created by the scan for the elements of the environment that don’t contain a lot of chrome (eg, vanity) [Diary – P1]*  *The wall behind the bed caused an error message […] because it [the wall] was single colored. [Diary – P2]*  *Bathroom too small, the resulting scan is not good for taking measurements. I had the error message but impossible to step back. [Diary – E2]*  *I had difficulty with some of my scans. My bathroom scan in my main bathroom did not work because I have a reflective floor. It is dark black but when it’s freshly cleaned, you can see yourself in it. The app did not like that. [Interview – E6]* |
| Image quality | Scan's visual quality and most common defects (holes, distortion, blurriness, duplication or confounded elements) | *If you really need a precision on the environment, the shape of the toilet, precision on the bath’s rim, I find the scan is not clear enough in a photographic way to tell me. [Interview – E5]*  *Honestly, I found the scan came out clear, […] I was looking at a patient’s scan and the physio entered the office at the same time and was impressed by the visual quality. [Interview – E7]*  *It’s kind of special, it looks like a room that went through a fire [Interview1 – P3]*  *The quality is good. [Interview3 – P1]*  *Bathrooms are often small and have many reflecting elements (shinny ceramic, chrome-plated elements, mirrors). This does not favor a scan of optimal quality. [Logbook – NST]* |
| Validity of measurements | Validity of measurements made on scans and desired precision | *He [patient] had given a measurement. It allowed me to compare and […] there were only a difference of a couple millimeters. [Interview – E5]*  *It allowed us to see that it’s not always reliable […] I think there was a difference of two inches in one place. For a toilet seat, it can make a big difference. But I think it’s a question of environment […] [Interview – E2]*  *Floor to ceiling height in MapIt vs manually are similar (near the shower MapIt : 2.13m vs me: 2.14m and total height MapIt: 2.77m vs me: 2.71m) [Diary – P2]*  *[…] sometimes, a centimeter makes the whole difference between a wheelchair that can pass or not through the doorway. […] Q: So for you, it would have to be precise enough, almost the same measurement as you would take with a measuring tape? A: Yes, that’s it. [Interview2 – P3]* |
